# Modulation of DNA Damage by Epstein–Barr Virus (EBV)

**DOI:** 10.3390/v18091035

**Published:** 2026-09-18

**Authors:** Emily DiMaulo-Milk, Blossom Damania

**Affiliations:** 1 Lineberger Comprehensive Cancer Center, University of North Carolina, Chapel Hill, NC 27599, USA; 2 Genetics and Molecular Biology Curriculum, University of North Carolina, Chapel Hill, NC 27599, USA; 3 Department of Microbiology & Immunology, University of North Carolina, Chapel Hill, NC 27599, USA

**Keywords:** Epstein–Barr virus (EBV), DNA damage response (DDR), EBNA1, EBNA2, LMP1, BZLF1, reactive oxygen species (ROS), Burkitt lymphoma, herpesviruses, oncogenic viruses

## Abstract

Epstein–Barr Virus (EBV) is an oncogenic gammaherpesvirus present in >95% of the adult population. EBV is responsible for nearly 2% of the global cancer burden and is a contributing factor in the development and progression of both epithelial and lymphoid malignancies. Throughout its lifecycle, EBV manipulates the DNA Damage Response (DDR). Aberrations in the DDR pathway result in genomic instability, a hallmark of cancer. In this review, we explore how EBV gene products expressed during lytic reactivation and latency promote genomic instability by creating a genotoxic environment and interacting with components of the DDR. Additionally, we describe viral genes capable of suppressing critical checkpoints which facilitate repair of damaged DNA. The ultimate failsafe in the case of catastrophic DNA damage is senescence or cell death, processes which are suppressed by EBV gene products. We also discuss how EBV appropriates DDR machinery to replicate its own genome during lytic reactivation. The manipulation of DDR proteins is essential for EBV-induced immortalization of primary B-cells infected in vivo, and viral strains engineered to lack these genes show reduced or ablated transformation efficacy, demonstrating the critical role of the DDR in EBV-induced oncogenesis.

## 1. Introduction

### 1.1. Epstein–Barr Virus (EBV)

In 1964, the gammaherpesvirus EBV, alternately known as HHV-4, was the first human tumor virus identified in Burkitt Lymphoma, a pediatric cancer of B-cell origin [1]. Further research demonstrated that EBV can contribute to the development of numerous cancers. EBV-associated malignancies encompass tumors of both lymphoid and epithelial origin. These include both Hodgkin and Non-Hodgkin Lymphomas, NK/T-cell Lymphomas, Gastric Carcinoma, and Nasopharyngeal Cancer [1,2,3]. EBV is a near-ubiquitous human pathogen, with upwards of 95% of adults carrying the virus [3]. It is a potent carcinogen that is responsible for 1.3–1.9% of all cancers [4]. It has also been associated with the development of numerous autoimmune diseases, such as Multiple Sclerosis and Lupus Erythematosus [5,6,7,8]. EBV is a large, enveloped dsDNA virus with a genome of approximately 170 kb [2]. Its genome contains sequences for >80 different proteins as well as dozens of non-coding RNAs [3]. Many viral proteins exhibit mimicry for human proteins, allowing the virus to manipulate endogenous pathways to its advantage [2,3,9]. EBV has a multilineage tropism and infects both epithelial and lymphoid cells. The virus is typically transmitted through saliva, and infection frequently occurs during childhood. Often, acute infection is asymptomatic, but it can also result in infectious mononucleosis when acquired in adolescence [3].

**Figure 1:**
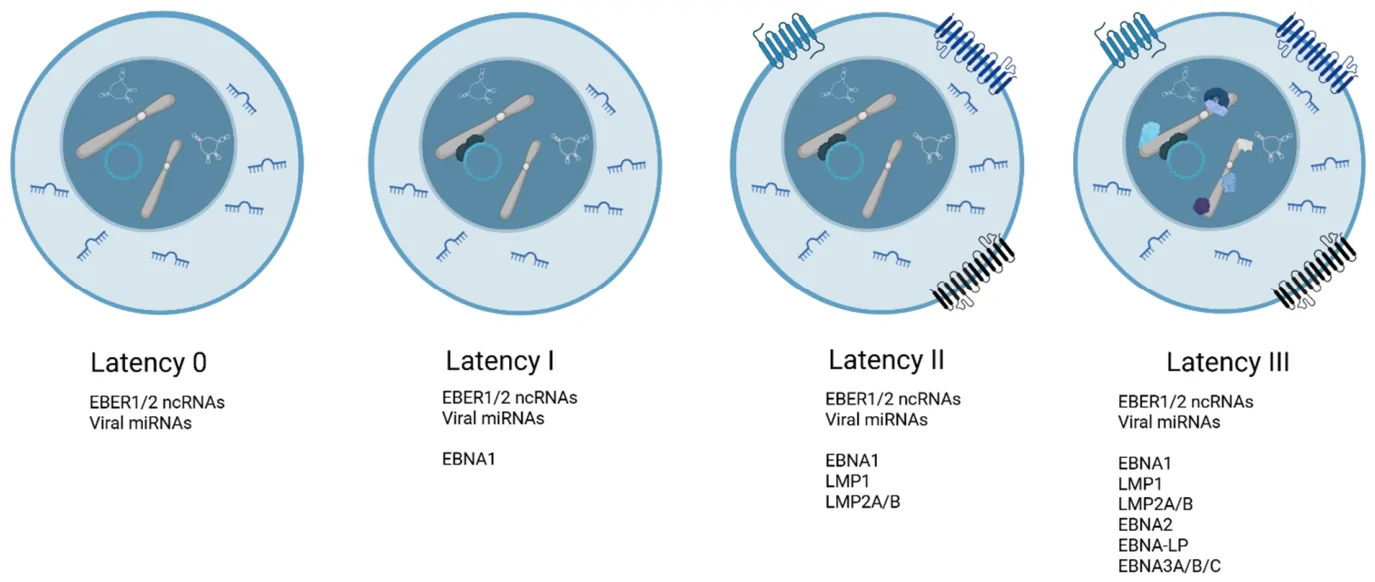
EBV latency patterns created in BioRender. DiMaulo-Milk, E. (2026) https://BioRender.com/sbolj3m.

Infection with EBV is lifelong, with the virus maintained in a subset of memory B-cells. EBV has a biphasic lifecycle characterized by the oscillation between lytic and latent infection. Its genome is linear in the viral particle but circular in latency. The lytic phase of EBV can be triggered by numerous conditions, a process termed reactivation [2,3]. The immediate-early viral proteins Zta (also known as BZLF1, ZEBRA, Z) and Rta (also known as BRLF1, R) initiate the transcriptional activation of the lytic cycle [3,10]. The viral genome is amplified 100- to 1000- fold during lytic reactivation, which is carried out by a viral DNA polymerase [2,3]. Ultimately, if successfully completed, the lytic phase of EBV results in the lysis of the host cell and release of infectious virus, enabling host-to-host transmission [3]. Partial reactivation can also occur, known as abortive lytic reactivation [11,12]. In abortive lytic reactivation, the expression of late lytic genes does not occur [3,11]. Therefore, EBV-infected cells can express immediate-early and early lytic genes but survive. The latent phase of EBV infection can be further subdivided into Latency 0/I/II/III (Figure 1). In healthy persons, EBV is usually in Latency 0, during which viral ncRNAs and miRNAs are detectable. During latency, there is limited to no production of viral proteins, with a maximum of nine viral proteins detectable during Latency III [3]. In Latency I, EBNA1 is expressed in the nucleus. During Latency II, additional transmembrane proteins LMP1, LMP2A, and LMP2B are detectable. Latency III results in the additional expression of the nuclear proteins EBNA2, EBNA-LP, and EBNA3A/B/C (Figure 1). Latent EBV is maintained as a circular chromatinized episome which is tethered to host chromosomes via the viral protein EBNA1 [3]. During cell division, EBNA1 mediates the recruitment of host replication licensing machinery and DNA polymerase to replicate the viral genome [3,13]. In this way, infection with EBV persists long after any overt symptoms subside.

**Figure 2:**
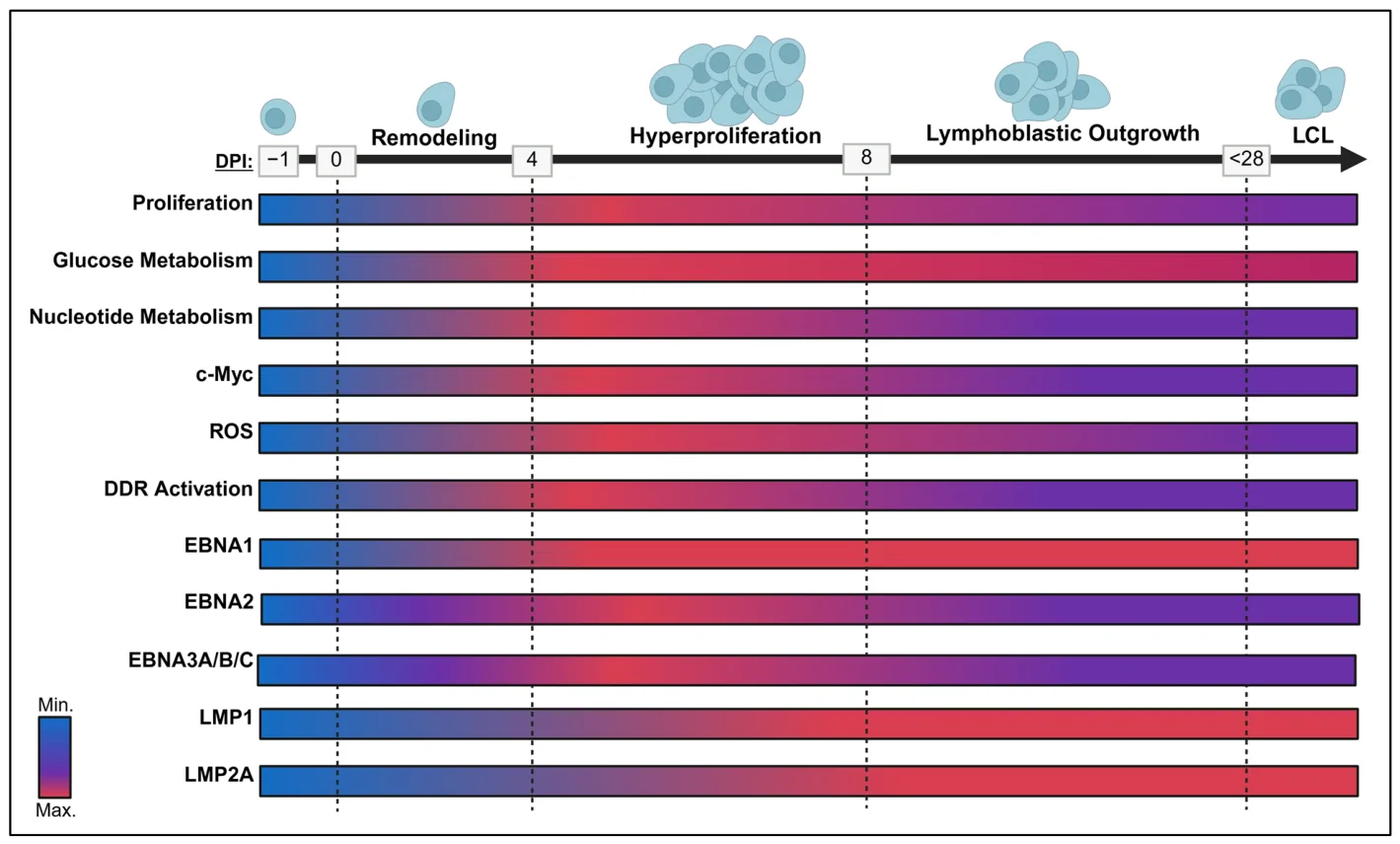
EBV-induced B-cell transformation results in the generation of LCLs. Ex vivo infection of isolated B-cells with EBV is sufficient to induce transformation of otherwise healthy cells into LCLs [15]. Approximately 1–10% of infected cells undergo immortalization [58]. The phases of transformation can be subdivided into at least three stages: remodeling from approximately 0–3 DPI, hyperproliferation during approximately 4–8 DPI, and LCL outgrowth as the cells transition into Latency III [59]. The precise timing of gene expression varies somewhat across reports, likely due to differences in host cells, viral strain, and other experimental conditions. This is a qualitative approximation of changes associated with EBV-induced B-cell transformation based on experimental results from numerous sources, as referenced here: proliferation [58,60,61], glucose metabolism [59,60], nucleotide metabolism [62], *c-Myc* [58,59], ROS [62,63], DDR activation [58,60,61,62,63], EBV gene expression [59,64]. Created in BioRender. DiMaulo-Milk, E. (2026): https://BioRender.com/ooopvtl.

EBV-positive tumors occur across multiple lineages and present in varied stages of latency (Table 1), though lytic genes also contribute to the oncogenic potential of EBV [3,12,14]. In vitro, EBV-infected B-cells are capable of undergoing spontaneous immortalization to form Lymphoblastoid Cell Lines (LCLs) (Figure 2) [15]. Expression of two latency proteins, LMP1 and EBNA2, is sufficient to transform primary B-cells in the absence of the viral genome, albeit less efficaciously [16]. Transgenic mouse models have demonstrated that expression of LMP1 alone results in lymphomagenesis [17]. Yet, this incredibly common virus causes cancer in a relatively small subset of those infected. People who are immunocompromised, such as those living with HIV, have higher rates of EBV-associated malignancies, demonstrating the importance of immune surveillance in preventing the emergence of viral tumors [3]. Numerous other host-intrinsic and extrinsic environmental risk factors have been identified, though the exact mechanisms underpinning EBV-induced oncogenesis remain undefined [3]. It is well-established that EBV gene products interact with components of the DNA damage response (DDR) to orchestrate its lifecycle and during the transformation process [3]. These interactions can promote genomic instability, a factor in tumorigenesis [3,18]. Here, we review the DNA damage response, how EBV induces DNA damage, and the impact of numerous viral gene products on processes related to genomic stability.

**Table 1:** Prevalence and expression pattern of EBV in associated diseases.

EBV-Associated Disease	EBV-Positive Cases [3,19]	Latency Pattern
All Cancers	1.3–1.9%	I/II/III
Hodgkin Lymphoma	30–40%	II
Burkitt Lymphoma	Endemic: >90% Sporadic: 20–50%	I
Diffuse Large B-Cell Lymphoma	9%	II/III
Post-Transplant Lymphoproliferative Disease	80%	III
NK/T-Cell Lymphoma	100%	II
Gastric Carcinoma	10%	I/II
Nasopharyngeal Carcinoma	80–100%	II

### 1.2. Induction of DNA Damage

During the lifespan of a cell, multiple kinds of DNA damage can occur due to distinct cell-intrinsic and extrinsic factors. The types of DNA damage include but are not limited to single-strand breaks (SSBs), double-strand breaks (DSBs), and DNA lesions, such as adducts or gaps, which impact bases but do not break the DNA backbone. The affected bases are themselves problematic and can promote strand breakage [20].

Of the types of DNA damage, DSBs are arguably the most catastrophic. This is because, in the absence of high-fidelity repair, DSBs can trigger cell death or generate oncogenic mutations [21,22]. DSBs can be induced by exogenous factors such as ionizing radiation or chemical insult [22,23,24]. Endogenously, DSBs can largely be attributed to issues during DNA replication [25,26]. As DNA polymerase runs along template DNA, the replication fork may stall. If the replication fork collapses or regresses, a DSB can be generated. Replication fork stalling can occur due to DNA damage (i.e., abasic sites, adducts, or interstrand DNA crosslinks) or independent of DNA damage (i.e., at repeat-rich regions, a collision with transcription complexes, depletion of free nucleotides, or R-loops) [25,26,27,28,29,30]. Interestingly, DSBs are more likely to occur on highly transcribed genes [25,27]. DSBs can also be generated through strictly regulated biological processes, notably during B/T-cell development to mediate V(D)J recombination [31].

SSBs and other DNA lesions where a base is damaged or absent but the DNA backbone is intact represent the majority of DNA damage events [20,32,33]. Replication fork stalling and spontaneous depurination/deamination creating an abasic site can cause SSB and DSB formation at a damaged or absent base [20,32]. Additional mechanisms of SSB generation include ionizing radiation and abortive activity of enzymes which induce nicks in DNA, such as TOP1 [20,32]. Intrastrand cross-links, a type of DNA damage, are induced exogenously by UV radiation and chemicals found in cigarette smoke or pollution [34]. Endogenous inducers of DNA adducts include metabolites such as Reactive Oxygen Species (ROS) and Nitric Oxide (NO), which cause oxidative DNA damage [33,34,35,36]. Lipid peroxidation can also generate bulky DNA adducts [37]. One of the most abundant and well-studied DNA adducts is ROS-derived 7,8 dihydro-8-oxoguanine (8-oxoG). While guanosine normally pairs with cytosine, 8-oxoG derived from guanosine instead pairs with adenine [33,34,35]. Other DNA adducts mispair similarly [33,37]. Endogenous errors in DNA replication which result in a base mismatch also generate DNA lesions [38]. The regulated generation of base mismatches occurs as a normal part of the adaptive immune response via the cytosine deaminase AID during the Germinal Center Reaction (GCR) in two distinct processes: Somatic Hypermutation (SHM) and Class-Switch Recombination (CSR) [39,40]. Overall, an estimated 70,000 DNA lesions are generated per day per cell in the human body [20].

### 1.3. The DNA Damage Response (DDR)

To ensure fidelity of the genome, cells must recognize and repair these diverse forms of DNA damage. This process is known as the DNA Damage Response (DDR) [23,41]. The DDR can be roughly distinguished into three phases: (i) recognition of damage; (ii) signal transduction; and (iii) removal of damage and resynthesis of DNA [41,42]. The components of these three stages are referred to as sensors, transducers, and effectors, respectively, though factors may contribute to more than one stage of the DDR [42]. The type and magnitude of DNA damage determine which components of the DDR are activated. The phase of the cell cycle also determines the response to DNA damage [43]. Cell cycle arrest is initiated to allow time for the repair process, and, in the presence of catastrophic, irreparable DNA damage, cells can initiate programmed cell death or senescence [29,43,44,45,46]. Defects in the DDR can result in the continued survival and replication of cells with catastrophic DNA damage, driving mutagenesis, which can contribute to the development and progression of cancer [46]. If cells proceed through the cell cycle in the absence of effective repair, loss of genomic integrity can occur, which is a hallmark of cancer [18,23,25,46,47]. Pathological silencing of tumor suppressors or activation of oncogenes can also indirectly contribute to genomic instability via dysregulation of origin licensing and induction of DNA damage through altered metabolism [29,48].

DSBs are repaired through one of three pathways: (i) Homologous Repair (HR); (ii) Non-Homologous End-Joining (NHEJ); or (iii) Theta Mediated End-Joining (TMEJ, Alt-NHEJ, MMEJ) (Table 2). HR is a high-fidelity repair process which relies on the presence of a sister-chromatid template and is thus restricted to the S/G2 phase [26]. Therefore, most DSBs are repaired via error-prone NHEJ. NHEJ is initiated via the binding of the Ku70/Ku80 heterodimer followed by recruitment of factors such as MDC1, RNF168, 53BP1, and DNA-PKcs [49,50]. The serine/threonine kinase, ATM, is a sensor and transducer which recognizes DSBs and phosphorylates itself, the histone subunit H2A.X to γH2A.X, and the transducer Chk2 [24,42]. Exposed ssDNA present during the DSB repair process can activate another DDR sensor and transducer, the serine/threonine kinase ATR, and its substrate, the transducer Chk1 [42].

Multiple repair pathways exist to repair damaged bases. In some cases, a DNA adduct can be removed from a base to reverse DNA damage [52]. However, in most cases, the damaged base must be removed for repair to occur. The excision of the damaged base generates an SSB, resulting in the activation of ATR/Chk1 [42]. Repair is carried out via one of three pathways: Mismatch Repair (MMR), Base Excision Repair (BER), or Nucleotide Excision Repair (NER). Both the sensor and preferred repair pathway depend upon the type of DNA lesion. Small DNA adducts can be identified via glycosylases, which traverse the genome scanning for adducts, such as OGG1, which recognizes 8-oxoG [53]. Repair is then mediated by the BER pathway (Table 3) [20]. Components of BER are used for SSB repair, but recognition of SSBs is performed by PARP1 rather than DNA glycosylases [32]. Base-mismatch and bulky DNA adducts such as those induced via UV radiation are repaired via MMR and NER, respectively (Table 3) [38]. In the absence of repair, translesion polymerases can be utilized to bypass a damaged or absent base. Translesion polymerases have affinity for specific substrates and generally incorporate the correct base but lack any proofreading capabilities. Outside of their specific substrate, translesion polymerases are substantially more error-prone and can thus contribute to genomic instability [54].

To allow repair of damaged DNA, the cell cycle must be paused [43]. The induction of cell cycle arrest, senescence, and/or apoptosis is mediated by ATR/ATM and their downstream effectors, Chk1 and Chk2 [42]. Transient activation of the DDR results in cell cycle arrest via inhibition of cyclin-dependent kinases, stabilization of p53, and upregulation of p21 [23,41,43]. At times, DNA damage may be so profound that endogenous repair pathways cannot re-establish genomic integrity. In these cases, the cell will usually undergo senescence or programmed cell death. The deterministic factors for arrest vs. death are complex and incompletely understood. Both p53-dependent and p53-independent means of cell death can occur [44,45,55]. The induction of p16, particularly in response to ROS and in the absence of functional p53, results in senescence [56]. Sustained activation and stabilization of p53 induces the transcriptional activation of pro-apoptotic effectors which inhibit the anti-apoptotic members of the Bcl-2 family. Subsequently, under normal conditions, apoptosis is initiated [23,44]. However, infection with EBV disrupts normal conditions. Indeed, viral genes expressed throughout the EBV lifecycle alter the DDR at every level.

**Table 3:** Mechanisms and components of DNA lesion and SSB repair.

Repair Pathway	Usage	Components	Fidelity
Mismatch Repair (MMR) [38]	Base-mismatch, Somatic Hypermutation (SHM), Class-Switch Recombination (CSR)	hMutSα, hMutSβ, PCNA, RPA, ATR/Chk1	High fidelity unless translesion polymerases are used and during SHM/CSR
Base Excision Repair (BER) [35]	Small adducts, abasic sites, oxidative damage (i.e., 8-oxoG), SHM, CSR, SSBs *	DNA glycosylases, (i.e., OGG1, MUTYH, UNG2), PARP1, APE1, PCNA, RPA, ATR/Chk1	High fidelity unless translesion polymerases are used and during SHM/CSR
Nucleotide Excision Repair (NER) [57]	Bulky DNA adducts, crosslinking, stalled RNAPII	XPC-Rad23B, CSB/CSA, TF_II_H, PCNA, RPA, ATR/Chk1	High fidelity unless translesion polymerases are used and during SHM/CSR
Transcriptional bypass [54]	Replication gaps, stalled replication forks, SHM, CSR, DNA adducts and abasic sites (unfavored)	Translesion polymerases (i.e., Pol θ, Pol η), PCNA, RPA, RAD18, ATR/Chk1	No proofreading, medium fidelity for specific substrates, low fidelity otherwise

* components of BER are used for SSB repair.

## 2. EBV-Induced DNA Damage

### 2.1. Induction of Reactive Oxygen Species

The increased consumption of glucose in cancer, known as the Warburg Effect, is connected to genome fidelity in numerous ways, primarily via the induction of DNA-damaging ROS [65]. EBV-positive lymphoma and epithelial cell lines show demonstrably higher accumulation of the oxidized DNA lesion, 8-oxoG, compared to coisogenic EBV-negative counterparts [66]. Research using the B-cell transformation model has provided substantial evidence that EBV infection results in a metabolic shift (Figure 2) [58,59]. During and following transformation, cells upregulate numerous glycolytic enzymes, consume more glucose, and generate substantially higher levels of lactate, a by-product of glycolysis, relative to uninfected cells [59,67]. Similarly, infection of B-cells with EBV results in greater accumulation of ROS relative to stimulated B-cells [63]. ROS levels peak during the hyperproliferative phase [63]. Lipid peroxidation also increases during transformation, with the highest levels during the hyperproliferative phase [68]. Accordingly, transformation increases the presence of the DNA damage and replication-stress marker, γH2A.X, with the highest detection during hyperproliferation [58,61]. Therefore, EBV infection evidently induces DNA damage indirectly through its effects on metabolism and the generation of ROS.

The primary EBV genes responsible for metabolic remodeling are LMP1 and EBNA2 [69]. Beyond enhancing glucose metabolism, these two viral genes have been implicated in numerous metabolic pathways [59,67,70]. Glucose metabolism is enhanced in both B-cells and epithelial cells when LMP1 is present [71,72,73]. LMP1 is a ligand-independent CD40 mimetic which is expressed as early as 4 days after infection and in Latency II/III (Figure 1 and Figure 2) [64]. It stimulates endogenous host pathways such as canonical and non-canonical NF-κβ, MAPK, and JAK/STAT signaling [64]. EBNA2 is expressed shortly after infection and in Latency III (Figure 1 and Figure 2) [74]. EBNA2 is essential for B-cell proliferation during the hyperproliferative phase [61]. In conjunction with chromatin remodelers, host transcription factors, and the viral protein EBNA-LP, EBNA2 modulates the expression of hundreds of host genes [74,75]. It also enhances the expression of LMP1 [74]. Strikingly, *c-Myc* is a direct target of EBNA2 [76,77]. *c-Myc* is a well-established oncogene and master regulator of metabolism which promotes glycolysis and lipid metabolism [78,79]. EBNA2-induced expression of *c-Myc* is highest during hyperproliferation, though it remains high during outgrowth (Figure 2) [58,59,69].

While LMP1 and EBNA2 are amongst the most potent metabolic remodelers expressed by EBV, they are far from the only viral proteins which promote ROS generation and genomic instability. EBV-positive cell lines in both Latency I and III generate more ROS and have higher levels of γH2A.X compared to genetically matched EBV-negative counterparts [80,81]. Further, Latency I and Latency III are both associated with markedly higher levels of chromosomal aberrations [81]. The only viral protein consistently expressed in Latency I, EBNA1, is critical for the maintenance and replication of the viral genome and is expressed in all EBV-positive tumors (Table 1 and Figure 1) [3,13]. It also modulates the expression of both viral and host genes, including those involved in metabolism [13,82]. Interestingly, expression of EBNA1 alone is sufficient to increase intracellular ROS, 8-oxoG, γH2A.X, and associated DSBs [66,82,83,84]. The expression of the DNA glycosylases, OGG1 and MUTYH, which recognize and excise oxidized DNA, is increased in the presence of EBNA1 [66]. EBNA1 also increases the expression of MTH1, an enzyme that removes oxidative damage from free nucleotides [66]. Altogether, EBV gene products expressed during early infection and in all EBV-positive tumors mediate metabolic changes indirectly linked to DNA damage [60,69].

### 2.2. Altered Nucleotide Metabolism and Associated Replication Stress

During the hyperproliferative phase, cells divide roughly every 8–12 h (Figure 1) [58]. Replication of these infected cells requires the availability of free nucleotides; however, the resting B-cells that EBV infects have low levels of nucleotide synthesis [79]. EBV gene products upregulate several factors involved in nucleotide metabolism. The expression of ADA and AK4, which contribute to adenosine generation, is markedly higher throughout transformation relative to uninfected cells [85]. EBNA1, EBNA2, and EBNA3C bind at the enhancers of these genes [85]. De novo pyrimidine synthesis is enhanced in the presence of EBV. During and following hyperproliferation, upregulation of genes involved in this process, UCK2, CTPS1, and CTPS2, occurs [86]. Loss of CTPS1 or CTPS2 results in increased γH2A.X, and co-depletion is substantially toxic to LCLs [86]. Overall, the transformation and survival of EBV-infected cells are dependent upon increased nucleotide generation following infection.

By targeting nucleotide metabolism, EBV gene products can abrogate the replication stress induced by viral infection. However, numerous lines of evidence demonstrate that replication stress and DNA damage still occur. Gene Ontology (GO) enrichment analysis of EBV-induced B-cell transformation has demonstrated a significant transcriptional activation of programs related to DNA replication and cellular response to DNA damage stimulus, which peaks during the hyperproliferative phase (Figure 2) [87]. γH2A.X levels increase concomitantly with the induction of DNA synthesis during this time [58,61]. The majority of γH2A.X signal co-localizes with host chromosomes rather than the viral genome [58]. Congruent activation of ATR, which responds to both replication stress and SSBs, occurs during hyperproliferation, while pharmacological inhibition of ATR abrogates the increase in γH2A.X [61,62]. EBV-positive cell lines also express higher levels of ATR and Chk1 relative to primary B-cells [88]. Exogenous supplementation of free nucleotides at infection enhances the transformative potential of EBV and reduces the abundance of ATR foci [62]. Interestingly, purine (A/G) supplementation alone was sufficient to generate this effect [62].

Replication stress can be induced by numerous factors, such as the presence of DNA lesions (i.e., those generated via oxidative damage from elevated ROS) and the depletion of free nucleotides [20,32]. While it was initially unclear if the observed activation of ATR was due to oxidative DNA damage or replication stress, later work elegantly resolved this conflict and demonstrated that EBV-infected cells experience DNA damage and replication stress during this time [58,61,62]. In fact, both DNA damage and replication stress are highest during the hyperproliferative phase of the transformation process [62]. Possibly, the EBV-induced glycolytic burst and the depletion of free nucleotides intersect to create a genotoxic environment during this time. Overall, viral gene products indirectly induce DNA damage and replication stress due to the activation of metabolic and proliferative pathways. Associated mutagenesis and the contribution of this effect to in vivo lymphomagenesis remain to be fully explored.

### 2.3. Aberrant Activation of B-Cell Developmental Programs

At certain stages in B-cell development, DNA damage is imparted via the carefully regulated expression of host proteins. This selective induction of DNA damage is critical to V(D)J recombination and CSR/SHM [31,39]. However, the type of DNA damage and its subsequent repair differ between these two developmental programs.

V(D)J recombination enables the generation of diverse T-and B-cell receptors. In brief, recombination occurs at the immunoglobulin (Ig) loci at recombination signal sequences (RSS). The lymphoid-specific proteins RAG1 and RAG2, in conjunction with other factors, cleave DNA at RSS to generate an asymmetric DSB which is favorably repaired through NHEJ, which induces mutations [31]. Inappropriate activation of RAG promotes hematopoietic malignancies [89,90,91]. RAG1-mediated cleavage at cryptic RSS is relatively rare, though downregulation of the DSB sensor/transducer, ATM, significantly increases the frequency of cryptic recombination and associated chromosomal rearrangements [92]. It is unclear whether components of V(D)J recombination are impacted by EBV infection. EBV preferentially infects mature CD21+ B-cells that have already undergone V(D)J recombination [3]. Research investigating the impact of EBV gene products on RAG expression has produced contradictory results, with some suggestions that EBNA1 may promote RAG expression [93,94,95,96]. Conversely, LMP1 may mediate a downregulation of the RAG proteins, possibly via repression of the transcription factor FOXO1, which normally activates RAG expression [97,98]. Ultimately, the relationship between EBV and V(D)J recombination remains largely unexplored.

In contrast, it is well-known that EBV gene products impact components of SHM and CSR. SHM is a developmental process which occurs as part of the GCR to enhance antibody affinity in activated B-cells, while CSR controls the antibody isotype [40]. Normally, the GCR is initiated in response to antigen binding at the BCR and co-stimulation from ligands expressed by T-cells/antigen presenting cells, namely CD40L (CD154) [99]. The EBV proteins LMP2A and LMP1 mimic a constitutively active BCR and CD40, respectively [3]. Accordingly, infection with EBV can enable even BCR-negative cells to undergo the GCR by inducing chromatin remodeling and phenotypic changes downstream of BCR stimulation [100,101].

Both SHM and CSR are mediated via AID [40]. AID deaminates cytosine residues present in ssDNA [102]. Accordingly, R-loops and actively transcribed genes are more vulnerable to AID-induced mutagenesis [103,104]. It is unclear how AID activity is regulated. In fact, AID broadly targets transcribed genes in healthy B-cells [105]. The majority of off-target deamination events are corrected with fidelity via either MMR or BER, but a significant proportion of actively transcribed genes can accumulate mutations during the GCR [105]. Deamination of cytosine generates a uracil, which is mispaired with guanosine [39,103]. The uracil can be removed by UNG2, which promotes BER, be repaired via MMR, or generate a DSB [39,106]. A mouse knockout of UNG2 has deficiencies in CSR and SHM, demonstrating its importance in this process [107]. Strikingly, EBV encodes its own uracil DNA glycosylase, BKRF3, which is an early-lytic gene [108,109]; however, it is unknown if BKRF3 acts upon the host genome.

Infection of B-cells with EBV induces the expression of AID and associated mutational signatures, presumably via LMP1 and/or EBNA-3C [110,111,112,113]. EBNA2 seems to curtail this effect [114]. AID can cause chromosomal rearrangements and is required for the formation of a *c-Myc* translocation, which defines Burkitt Lymphoma [115]. Notably, tumors of the endemic and sporadic forms of Burkitt Lymphoma are over 90% and 20% EBV-positive, respectively (Table 1) [3,19]. It is also worth emphasizing that the transcriptional induction of *c-Myc* occurs upon EBV infection, which may increase the availability of the ssDNA substrate of AID at the *c-Myc* loci [69,76,77]. Interestingly, lytic replication of the EBV genome involves R-loop formation, a feature which is vulnerable to off-target AID [116]. It remains unanswered whether the EBV genome is vulnerable to AID-induced mutagenesis.

### 2.4. Direct Induction of DNA Damage

In addition to the indirect impacts of modulating intrinsic host pathways, there are also cases where the direct physical interaction of viral proteins with the host genome seemingly causes DNA damage. Zta is an immediate-early lytic gene that mediates the transcriptional activation of the lytic cascade by binding to Z-responsive elements in the EBV genome [3]. While EBV-positive cancers consist largely of cells in latency, Zta has been detected in tumors, and the lytic phase contributes to tumorigenesis in vivo [14,117,118,119]. Thousands of Zta binding sites along the host genome have been detected in both epithelial and lymphoid cells, and a significant proportion of these binding sites are conserved between the two lineages [120,121,122]. Zta is homologous to the host transcription factor AP-1 [120]. It has pioneer activity; that is, it triggers extensive, genome-wide chromatin remodeling [122]. Pseudotime analysis of cells undergoing spontaneous lytic reactivation further supports the pioneering function of Zta [123]. Expression of Zta in isolation results in a significant increase in pATM and γH2A.X, which is dependent upon the Zta DNA-binding domain [124]. Single-cell resolution analysis demonstrates a positive correlation between Zta expression and γH2A.X as well as enrichment for pan-nuclear γH2A.X in Zta-positive cells [123]. GO enrichment analysis did not identify DDR or DNA synthesis among the top enriched biological processes in Zta-regulated host genes, suggesting that its DNA-damaging effect is not due to a transcriptional suppression of DDR machinery [120]. The precise mechanism of Zta-induced DNA damage remains controversial. However, γH2A.X distribution is not exclusive to Zta binding sites, but rather pan-nuclear [123]. Therefore, it is likely that broad changes in chromatin architecture due to pioneering activity of Zta are at least somewhat responsible for the induction of DNA damage.

BGLF5 is an early lytic gene which codes for an exonuclease with both DNase and RNase activity [125,126]. During early lytic reactivation, BGLF5 degrades host mRNAs to suppress the immune response [125]. It also plays an important role in the processing of the viral genome for packing into viral particles, namely cleaving the circular viral genomes into a linear form [126]. BALF3 is a lytic gene with similar nuclease activity [2]. Both BGLF5 and BALF3 have been implicated in genomic instability in epithelial cells, which may be due to post-transcriptional suppression of DDR mRNAs or nuclease activity on the genome itself [127,128].

Expression of EBNA1 is sufficient to increase chromosomal abnormalities in an EBV-negative background [84]. Recent work has identified that EBNA1 binding is substantially enriched at a fragile site in chromosome 11q23 [129]. This site is vulnerable to breakage and micronucleation when EBNA1 is expressed ectopically or in the presence of latent EBV [129]. The likelihood of a break positively correlates with EBNA1 expression [129]. Further, mitotic chromosomal rearrangements occur at this site, which are then inherited by daughter cells [129]. Therefore, expression of EBNA1 can generate heritable oncogenic chromosomal rearrangements which would endure even if the viral episome was lost. In this way, the oncogenic impact of EBV could be a “hit-and-run”: the development of cancer is caused by EBV, but the tumor is EBV-negative upon diagnosis.

## 3. Effects of Viral Proteins on Components of the DDR

### 3.1. Inappropriate Cell-Cycle Progression and Suppression of Apoptosis

Ultimately, the DDR concludes with cell cycle arrest to facilitate repair, followed by activation of senescent or apoptotic programs in the case of sustained damage [43,44]. EBV disrupts this process, as numerous lytic and latent genes inhibit the activation of apoptotic pathways [3,130]. Some of the viral genes which inhibit p53 include Zta, EBNA1, EBNA3C, LMP1, and miR-BART5-3p [124,131,132,133]. Additional viral proteins promote the progression of the cell cycle indirectly via *c-Myc* activation, enabling evasion of checkpoints that are essential for genomic stability [76,134]. Other viral genes manipulate members of the anti-apoptotic Bcl-2 family either directly or indirectly [135,136,137]. Recent work has identified a novel role for EBV proteins in modulating the expression of NEK2, a component of the mitotic checkpoint which has been implicated in numerous cancers [138]. EBNA1, EBNA2, and LMP1 each independently upregulate NEK2 [137,139]. Overexpression of NEK2 is associated with aberrant cell cycle progression and chromosomal instability [138]. Additionally, NEK2 reportedly destabilizes p53, and the anti-apoptotic proteins Bcl-2, Mcl-1, and Bcl-xL are modulated by NEK2 to promote the survival of EBV-positive lymphomas [137,138]. The impact of EBV on NEK2 expression is an excellent example of how the DDR is manipulated, from the induction of DNA damage to inappropriate progression of the cell cycle and the ultimate suppression of apoptosis. Through divergent means, EBV gene products enable the growth and survival of cells, even those which are pre-malignant.

### 3.2. The DDR in Lytic Reactivation

In addition, various viral proteins modulate components of the DDR itself. The role of the DDR in the EBV lifecycle is complex, with components alternatively supporting or suppressing the virus dependent upon the phase of infection.

ATM activation promotes the lytic reactivation of EBV [140]. Despite the increase in ATM activation, arrest or death does not occur, and expression of cyclin-dependent kinases during lytic reactivation is comparable to S-phase [141]. Numerous viral proteins suppress the recognition and repair of DSBs during lytic reactivation. BKRF4 is a tegument protein contained within the viral particle which is also expressed during early lytic reactivation [142]. BKRF4 inhibits histone ubiquitination at the site of a DSB by interfering with the E3 ubiquitin ligase RNF168 and subsequent 53BP1 recruitment, thus suppressing NHEJ [142,143]. The early lytic gene BMRF1 (Ea-D), conventionally involved in viral genome replication, also inhibits the recruitment of RNF168 during lytic reactivation [144]. Zta suppresses the recruitment of 53BP1 [124]. In this way, while activation of ATM supports lytic reactivation by promoting the virus’s entry into this phase, the culmination of the associated DDR is suppressed to enable completion of lytic reactivation.

The tegument and early lytic gene, BGLF4, is a serine/threonine protein kinase, which phosphorylates and inactivates host DNA helicase [145,146]. BGLF4 further induces premature condensation of host DNA [147]. Resultingly, expression of BGLF4 suppresses the G1/S transition and delays S-phase, resulting in chromosomal aberrations, polyploidy, and micronuclei formation [148]. BGLF4 has also been demonstrated to interact with DDX5/17 [149]. Both DDX5 and DDX17 have been implicated in the repair of R-loops, DNA/RNA hybrids, which promote DSBs and genomic instability [150,151]. Loss of DDX5/17 results in significantly reduced Zta expression and blocks lytic reactivation [149].

BPLF1, a tegument and late lytic gene, deubiquitinates PCNA, which localizes to sites of replication stress [152,153]. When ubiquitinated, PCNA promotes the bypass of DNA lesions [152]. A reduction in ubiquitinated PCNA is associated with attenuated localization of the translesion polymerase, Pol η, at sites of DNA damage [152]. BPLF1 stabilizes the E3 ubiquitin ligase that targets PCNA, Rad18, during lytic replication, which enhances the generation of infectious virus [154,155]. Interestingly, both PCNA and Pol η support the generation of infectious virus during lytic reactivation, and Pol η preferentially binds to the viral genome over the host genome at this time [152,154]. Pol η is also upregulated by BPLF1 [154]. EBV may co-opt Pol η, driving it to eschew the host genome for that of the viral genome during lytic reactivation. It is speculated that recruitment of Pol η allows for the effective bypass of bulky DNA adducts in the viral genome, which could otherwise damage or block the replication of the viral genome [154]. Overall, during lytic reactivation, the virus suppresses the DDR at the host genome while simultaneously co-opting its elements to orchestrate viral replication.

### 3.3. Modulation of DSB Recognition and Repair

Latency genes also modulate the expression and activity of components of the DDR. As previously described, ATM is the sensor which recognizes DSBs and initiates a signaling cascade that facilitates DSB repair via Chk2 (Table 2). Isolated expression of the viral protein LMP1 in B-cell lines represses the expression of ATM [84,156]. This occurs at the transcriptional level and is mediated by LMP1-induced upregulation of a component of the polycomb-repressive complex, Bmi-1 [84,156]. In nasopharyngeal carcinoma cell lines, the presence of EBV is associated with the downregulation of ATM [157]. Viral miRNAs evidently contribute to the downregulation of ATM in this context [158]. During the hyperproliferative phase of EBV-induced transformation, increased activation of ATM and Chk2 occurs (Figure 2) [58]. Yet, the activation of ATM and Chk2 suppresses EBV-induced immortalization. Cells that arrest and thus fail to immortalize have higher expression of ATM targets and 53BP1 [58,60]. Application of either an ATM or Chk2 inhibitor substantially increases the transformation efficacy of EBV [58]. This effect is specific to the hyperproliferative phase, as application of these inhibitors after this window has passed has little to no effect [58].

The viral proteins EBNA3A and EBNA3C suppress the ATM-responsive signaling cascade [58,84]. Both proteins are expressed shortly after primary infection and in Latency III (Figure 1 and Figure 2) [58]. These proteins compete at EBNA2 binding sites and interact with endogenous host repressive complexes to downregulate gene transcription [3]. EBNA3C contributes to lymphomagenesis in vivo [159]. EBNA3C may physically interact with Chk2 to suppress its activity [160,161]. Further, EBNA3C acts in conjunction with EBNA3A to transcriptionally repress p16^INK4a^ [162,163,164]. Bmi-1 is also a negative regulator of p16, and previous work has suggested that LMP1 suppresses senescence via downregulation of p16 [56,165]. Additionally, EBNA2-induced expression of *c-Myc* peaks at the hyperproliferative phase (Figure 1) [58,59,69]. *c-Myc* also suppresses the expression of p21, which, in the context of the DDR, mediates cell cycle arrest to facilitate repair [78]. p16-induced senescence is an important secondary system in the absence of functional p53. Considering multiple viral gene products disrupt p53, the shutdown of p16 disables the senescence failsafe, while p21 suppression enables continued growth, allowing the proliferation of infected cells at a danger to the host. Revealingly, cells which fail to transform at the hyperproliferative phase have lower levels of EBNA3C and higher levels of p16, p21, and p53 [60]. Therefore, outside of lytic replication, activation of the DSB-associated DDR negatively impacts B-cell transformation.

## 4. Conclusions

As detailed in this review, numerous EBV genes target host factors involved in the DDR (Table 4). Additionally, distinct viral factors frequently target the same host elements. In part, this is likely attributable to the varied expression of viral genes throughout the EBV lifecycle, though latency genes are frequently co-expressed in EBV-positive tumors. Compounding the impact of EBV modulation of the DDR, there are multiple avenues by which viral gene products contribute to the generation of an environment which promotes DNA damage. This includes the increased generation of ROS, consumption of free nucleotides, and physical stress on the host genome. EBV-positive cells have higher levels of DNA damage markers, oxidized nucleotides, and genomic instability. EBV parasitizes elements of the DDR to trigger the lytic phase of its lifecycle and replicate its own genome. Taken together, the modulation of DDR components promotes the survival of cells during early infection, lytic reactivation, and latency in the presence of DNA damage that would otherwise induce senescence or death. While this is advantageous to the virus, there are negative impacts on the host cell which may translate to the development of an EBV-positive malignancy.

In addition to promoting the development of cancer, genomic instability also underlies treatment resistance [166]. However, limited research has been conducted evaluating the evolution of EBV-positive tumors. It is a distinct possibility that the presence of EBV could create greater intratumoral heterogeneity. Already evident is that, within a cell line or tumor, there exists heterogeneity in viral gene expression, such as the sporadic detection of Zta [119]. Activation of the DDR and loss of genomic integrity can trigger an anti-cancer immune response [23]. EBV-positive malignancies are more prevalent in people who are immunocompromised, suggesting that, in most people, an intact immune system would prevent the development of EBV-associated cancer via immune control of EBV-infected cells and/or the initiation of an anti-tumor response when oncogenic DNA damage occurs [3]. In vitro, it is well demonstrated that B-cells which undergo EBV-induced transformation are those that enhance glycolysis while suppressing the DDR, cell cycle arrest, and apoptosis. Yet, the contextual factors which drive the adoption of this state have not been elucidated. Additionally, research in this area has been largely restricted to in vitro models of EBV-associated cancers. Further investigation into the relationship between EBV, the DDR, and oncogenesis may help answer the question of how a near-universal virus does not universally cause tumors.

## Figures and Tables

**Table 2:** Mechanisms and components of DSB repair.

Repair Pathway	Usage	Components	Fidelity
Homologous Repair (HR) [26]	S/G2 phase, when the sister chromatid is present	Mre11/Rad50/Nbs1, BRCA1, BRCA2, Rad18, ATM/Chk2	High fidelity
Non-Homologous End Joining (NHEJ) [50]	Most DSBs, V(D)J recombination, Somatic Hypermutation, Class-Switch Recombination	Ku70/Ku80, MDC1, RNF168, 53BP1, DNA-PKcs, Artemis, ATM/Chk2	Low fidelity, mutagenic
Theta-Mediated End Joining (TMEJ) [51]	Broken HR or NHEJ, 2–20 bp microhomology	PARP1, Polθ	Error-prone

**Table 4:** Context and mechanisms of EBV gene products implicated in DNA damage and response. Cells are exposed to tegument proteins immediately following EBV infection. The immediate-early and early lytic genes can be expressed during abortive lytic reactivation. Additionally, leaky expression of numerous lytic genes has been reported.

EBV Gene	Expression Pattern	Implications in Genomic Instability	
		Increased	Decreased
EBNA1	Latency I, II, III	ROS, 8-oxoG, γH2A.X, DNA glycosylases, nucleotide metabolism, chromosomal aberrations, fragile site breakage, NEK2	p53
EBNA2	Latency III	*c-Myc*, glycolysis, ROS, γH2A.X, nucleotide metabolism, chromosomal aberrations, NEK2, associated with pATR, pATM, and pChk2	AID, cell cycle checkpoint
EBNA3A	Latency III		p16^INK4a^
EBNA3C	Latency III	AID	p16^INK4a^, Chk2
LMP1	Latency II, III	glycolysis, ROS, γH2A.X, chromosomal aberrations, NEK2, AID	p53, ATM, p16
LMP2A	Latency II, III	*c-Myc*, survival independent of BCR	cell cycle checkpoint
miR-BART5-3p	Continuous		p53
Zta	Immediate-early lytic	γH2A.X, pATM	p53, 53BP1 recruitment
BGLF4	Tegument, early lytic	chromosomal aberrations	host DNA synthesis
BGLF5	Early lytic	Unclear, possibly exonuclease activity on host genome	Unclear, possible reduction in DDR gene translation
BALF3	Late lytic	Unclear, possibly exonuclease activity on host genome	Unclear, possible reduction in DDR gene translation
BKRF4	Tegument, early lytic		53BP1 recruitment, RNF168 recruitment
BMRF1 (Ea-D)	Early lytic		53BP1 recruitment, RNF168 recruitment
BPLF1	Tegument, late lytic	Rad18, Pol η, Pol η recruitment to viral genome	Ub-PCNA and Pol η recruitment to host genome

## Data Availability

No new data were created or analyzed in this study. Data sharing is not applicable to this article.
